# Induction of cancer-associated fibroblast-like cells by carbon nanotubes dictates its tumorigenicity

**DOI:** 10.1038/srep39558

**Published:** 2016-12-20

**Authors:** Sudjit Luanpitpong, Liying Wang, Vincent Castranova, Cerasela Zoica Dinu, Surapol Issaragrisil, Yi Charlie Chen, Yon Rojanasakul

**Affiliations:** 1 Siriraj Center of Excellence for Stem Cell Research, Faculty of Medicine Siriraj Hospital, Mahidol University, Bangkok 10700, Thailand; 2 Pharmaceutical and Pharmacological Sciences Program, West Virginia University, WV 26506, USA; 3 Allergy and Clinical Immunology Branch, National Institute for Occupational Safety and Health, Morgantown, WV 26505, USA; 4 Department of Chemical Engineering, West Virginia University, WV 26506, USA; 5 Natural Science Division, Alderson Broaddus University, Philippi, WV 26416, USA; 6 West Virginia University Cancer Institute, West Virginia University, WV 26506, USA

## Abstract

Tumor microenvironment has been recognized as a key determinant of tumor formation and metastasis, but how tumor microenvironment is affected by nanomaterials is essentially unknown. Here, we investigated whether carbon nanotubes (CNTs), a widely used nanomaterial with known carcinogenic potential, can affect cancer-associated fibroblasts (CAFs), which are a key component of tumor microenvironment that provides necessary support for tumor growth. We show for the first time that single-walled CNT and to a lesser extent multi-walled and its COOH-functionalized form induced CAF-like cells, which are non-tumorigenic in animals, but promote tumor growth of human lung carcinoma and CNT-transformed lung epithelial cells. The mechanism by which CNT-induced CAF-like cells promote tumor growth involved the acquisition of cancer stem cells (CSCs) in cancer population. Gene knockdown experiments showed that an expression of podoplanin on CAF-like cells is essential for their effects, indicating the functional role of CAF-like cells and podoplanin in CNT tumorigenic process. Our findings unveil a novel mechanism of CNT-induced carcinogenesis through the induction of CAF-like cells that support CSCs and drive tumor formation. Our results also suggest the potential utility of podoplanin as a mechanism-based biomarker for rapid screening of carcinogenicity of CNTs and related nanomaterials for their safer design.

Due to their extremely small size, engineered nanomaterials (ENMs) can become airborne, be inhaled, and reach the pulmonary alveoli of the lungs. A major class of ENMs is carbon nanotubes (CNTs), which have increasingly been used for a wide variety of applications in fields as diverse as electronics, energy storage, waste treatment, consumer products, and medicine^1^,^2^. With such widespread uses, human exposure is to be expected during manufacturing, incorporation into products, product use and disposal^3^. Consequently, it is important to determine their unintended consequences, especially on human health and the environment. CNTs share several properties (e.g., high aspect ratio and biopersistence) and route of exposure (e.g., inhalation) with asbestos, a known human carcinogen. Therefore, concern has been raised for the possibility that CNTs would induce an asbestos-like lung cancer or mesothelioma risk^4^,^5^,^6^. Several animal studies have indicated the direct and indirect carcinogenic effects of CNTs, i.e., a single aspiration of single-walled (SW) CNT accelerated metastatic growth of lung carcinoma in the mouse model of tumor progression^7^,^8^, while subacute (15-day) inhalation of multi-walled (MW) CNT (Mitsui-7) promoted lung adenocarcinoma in the multi-carcinogenesis mouse model^9^. MWCNT have also been reported to induce mesothelioma after an intraperitoneal or scrotal injection^10^,^11^,^12^.

*In vitro* models have been developed to facilitate high-throughput screening of nanomaterial carcinogenicity and to aid detailed mechanistic investigations of their pathologic effects. Examples of such models include those that measure nanomaterials’ ability to malignantly transform cells and to induce cancer stem cells or stem-like cells (CSCs) upon chronic exposure^13^,^14^. Based on our current knowledge and emerging evidence on the role of the tumor microenvironment in tumor development^15^,^16^,^17^, we hypothesized that nanomaterials such as CNTs may induce changes in the tumor microenvironment that support tumor growth. Therefore, we investigated the effect of CNTs on cancer-associated fibroblasts (CAFs), a key component of the tumor microenvironment known to regulate tumor growth^18^,^19^. Because of their importance in tumorigenesis and metastasis, CAFs have been investigated as a novel target of cancer therapy and as a key contributor of the carcinogenic effect of various agents.

We reported herein that acute exposure of CNTs is capable of activating normal lung fibroblasts to become CAF-like cells, which have the propensity to promote tumor growth of human lung carcinoma cells and experimentally generated CNT-transformed lung epithelial cells through the mechanisms that involve CSC induction. With the rapid increase in the utility of nanomaterials^20^,^21^ and the lack of specific pre-screening tests for nanomaterial carcinogenicity, we also attempted to develop rapid, mechanism-based, test models and biomarkers based on their ability to induce CAFs and promote tumorigenesis. Podoplanin was identified as a key protein responsible for the tumor-promoting effect of CNT-induced CAFs and thereby could be a novel candidate biomarker for initial screening of the carcinogenicity of CNTs and related nanomaterials.

## Results

### CNT preparation and dose calculations

All CNTs used in the present study, including SWCNT, MWCNT and carboxylate (COOH)-functionalized (f)-MWCNT, were obtained from Cheap Tubes Inc. (Brattleboro, VT) in order to minimize the background differences in source materials and synthesis methods among samples and their certain physicochemical properties are summarized in Table 1. SWCNT and MWCNT were investigated in this study because they are the two major types of CNTs, while f-MWCNT may have wider applications due to its functionality. Prior to use, all CNTs were dispersed in 5% bovine serum albumin (BSA)-containing medium by water-bath sonication with the 130 W power, 20 kHz frequency, and 60% amplitude for 10 s as previously described^22^. Supplementary Figure S1 shows representative scanning electron microscope (SEM) micrographs of the dispersed CNTs and solid state Fourier transform infrared spectroscopy (FTIR) spectra of pristine MWCNT and f-MWCNT.

The CNT doses used in the *in vitro* exposure studies were calculated based on *in vivo* exposure data normalized to the alveolar surface area in mice and humans^23^,^24^. For example, the dose that induced positive *in vivo* biological response was 40 μg/mouse lung, which corresponds to 2 mg/kg body weight^22^,^25^,^26^. Dividing this dose by the average alveolar surface area in mice (~500 cm^2^)^27^ indicates the *in vitro* CNT surface area dose of 0.08 μg/cm^2^, which is equivalent to a human lung burden for 8 h/day over 2 years at 400 μg/m^3^ (high CNT level reported in a research facility)^28^ or about 76 years at 10 μg/m^3^ (average CNT level in U.S. facilities)^29^.

### Effects of CNTs on growth and transformation of primary human lung fibroblasts

**Figure 1:**
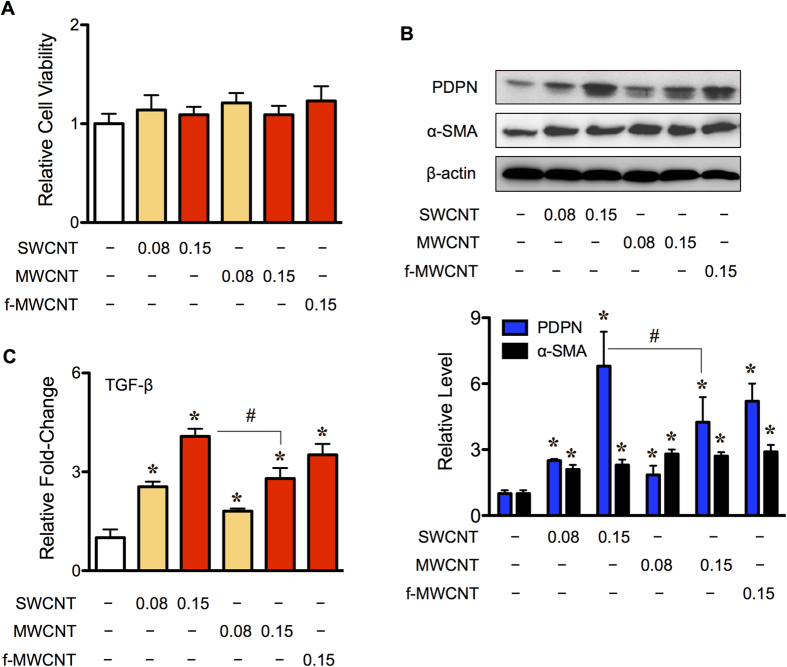
Carbon nanotubes induce transformation of primary human lung fibroblasts into cancer-associated fibroblasts. Subconfluent monolayers of human lung fibroblasts were treated with various concentrations (0–0.15 μg/cm^2^) of SWCNT, MWCNT, or f-MWCNT for 48 h. (**A**) Analysis of cell viability and proliferation using the WST-1 assay. (**B**) Western blot analysis of the cancer-associated fibroblast marker podoplanin (PDPN) and myofibroblast marker α-SMA. β-actin was used to confirm equal loading of the samples. Immunoblot signals were quantified by densitometry, and mean data from three independent experiments (one of which is shown here) were normalized to the results obtained in cells without CNT treatment (control). (**C**) Secreted TGF-β levels in the culture medium of CNT-treated lung fibroblasts as determined by ELISA. Data are mean ± SD (*n* = 3). *P < 0.05 *vs*. vehicle-treated control cells. ^#^*P* < 0.05 *vs* SWCNT-treated cells.

CAFs are a key component of the tumor microenvironment that provides the necessary support for tumor growth^18^,^19^. To test whether CNTs could induce CAFs and promote tumor formation, we investigated the effect of CNTs on human lung fibroblasts (LFs) with respect to their ability to induce CAFs and to support tumor growth induced by human lung carcinoma cells and CNT-transformed lung epithelial cells. Since previous studies have shown that CSCs are a key driver of tumor formation, and since our recent studies have shown that CNTs could trigger normal lung epithelial cells to initiate CSCs with high tumorigenic potential^13^,^14^, we also tested the effect of CNT-induced CAF-like cells on CSC formation. Primary human LFs were first treated with various concentrations (0–0.15 μg/cm^2^) of CNTs to determine their general cellular responses and viability using the WST-1 assay. Figure 1A shows that all of the CNTs tested including SWCNT, MWCNT and f-MWCNT had no significant effects on cell proliferation and viability at the concentrations used. These results were confirmed by CyQuant^®^ cell proliferation assay (Invitrogen, Carlsbad, CA) (data not shown). To test whether CNTs can induce CAFs, the expression levels of known CAF markers, including podoplanin and α-smooth muscle actin (α-SMA), a prototypic marker of myofibroblasts known to be enriched in CAFs^19^,^30^,^31^, were examined in CNT-treated human lung fibroblasts using Western blot analysis. Figure 1B shows that SWCNT, and to a lesser extent MWCNT and f-MWCNT, induced the CAF markers podoplanin and α-SMA. Additionally, the increase in secreted TGF-β, which represents a major cytokine released from CAFs^32^,^33^, was observed in response to CNT treatment (Fig. 1C) in concomitant with the upregulation of podoplanin, suggesting that CNTs were able to induce CAF-like cells, the finding that is supported by subsequent studies on tumor formation.

### CNT-induced CAF-like cells promote tumor formation

**Figure 2:**
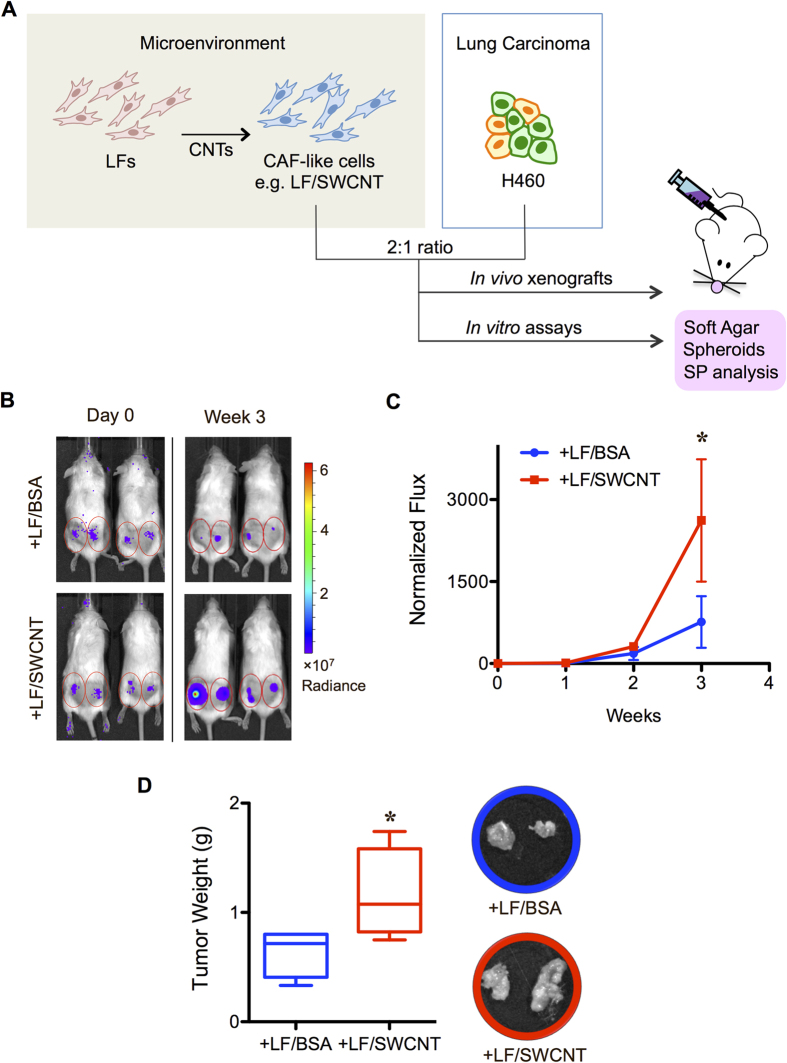
Carbon nanotube-induced cancer-associated fibroblast-like cells promote tumor formation of human lung carcinoma H460 cells. (**A**) Schematic diagram depicting methodological steps of *in vivo* and *in vitro* co-culture experiments. (**B**) SWCNT-induced cancer-associated fibroblast-like cells (LF/SWCNT) or vehicle-treated fibroblasts (LF/BSA) at the dose of 6 × 10^5^ cells were co-injected with luciferase-labeled H460 cells at the dose of 3 × 10^5^ cells (2:1 ratio) into the left and right flanks of NSG mice. Tumor formation was monitored weekly by IVIS bioluminescence imaging. IVIS images of mice at day 0 and week 3 are shown. (**C**) Normalization of tumor bioluminescence signals at various time points post-injection to their initial signals at day 0. (**D**) Subcutaneous tumors were dissected from mice bearing H460 and LF/SWCNT or LF/BSA, and weighted at the end of experiments at 3 weeks post-injection. Data are mean ± SD (*n* = 4). *P < 0.05 *vs* H460 and LF/BSA group.

It has been shown in the previous study^19^ and validated in the present study that co-injection of untreated LFs could promote the growth of human lung carcinoma xenograft tumors *in vivo* (Supplementary Figure S2A). To investigate the tumor-promoting effect of CAF-like cells induced by CNTs, we co-injected mice subcutaneously (SC) with CNT-induced CAF-like cells (LF/SWCNT) or with vehicle-treated lung fibroblasts (LF/BSA) and human lung carcinoma H460 cells at 2:1 ratio, as schematically depicted in Fig. 2A. To aid quantitation of tumor formation and growth, the lung carcinoma H460 cells were genetically modified to express luciferase (LUC2) for highly sensitive and non-invasive detection of tumor growth by bioluminescence imaging (IVIS^®^ Lumina II, PerkinElmer, Waltham, MA). Tumor luminescence signals were quantified over time and normalized to their initial signals at the time of inoculation (day 0). Figure 2B and C shows that tumor luminescence was substantially higher in the mice bearing H460 cells with LF/SWCNT cells, as compared to the mice bearing H460 cells with control LF/BSA cells, indicating the tumor-promoting effect of CNT-induced CAF-like cells. Direct measurements of tumor weight at the end of experiments (week 3) similarly indicated a marked increase in the excised tumor weight in mice treated with H460 cells and LF/SWCNT cells as compared to control treatment (Fig. 2D).

**Figure 3:**
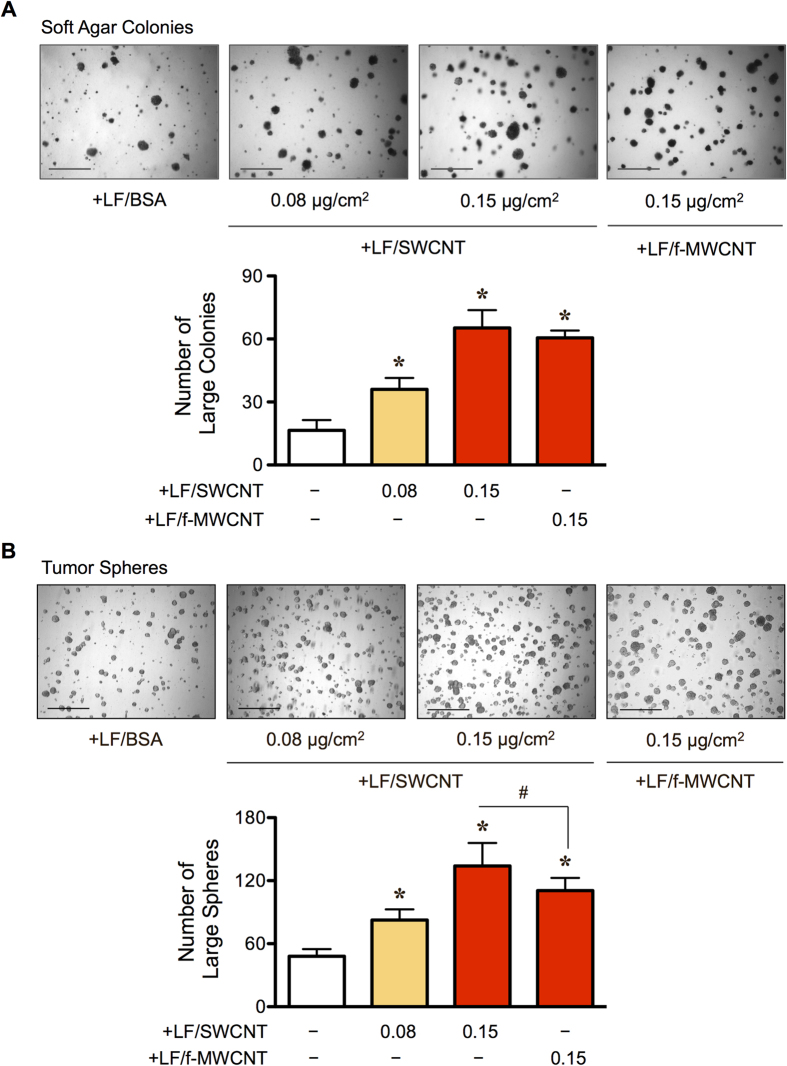
Carbon nanotube-induced cancer-associated fibroblast-like cells promote the growth and maintenance of cancer stem cells. Carbon nanotube-induced cancer-associated fibroblast-like cells (LF/SWCNT or LF/f-MWCNT) or vehicle-treated fibroblasts (LF/BSA) were co-cultured with GFP-labeled lung cancer H460 cells (2:1 ratio). (**A**) Analysis of soft agar colonies after 2 weeks of culture. Scale bar = 200 μm. (**B**) Analysis of tumor sphere formation after 2 weeks of culture. Scale bar = 300 μm. Data are mean ± SD (*n* = 4). *P < 0.05 *vs* H460 and LF/BSA group. ^#^*P* < 0.05 *vs* H460 and LF/SWCNT group.

To test whether CNT-induced CAF-like cells could similarly promote tumor cell growth *in vitro*, we co-cultured CNT-induced CAF-like cells with lung carcinoma H460 cells, as schematically depicted in Fig. 2A, and evaluated for their colony growth under soft agar assay, which is the most stringent indicator for malignant transformation associated with anchorage independence. Figure 3A shows that larger colonies were observed in the presence of SWCNT-induced CAF-like cells in a dose-dependent manner as well as in the presence of f-MWCNT-induced CAF like cells. These results strongly support the tumor-promoting effect of CNT-induced CAF-like cells.

### CNT-induced CAF-like cells promote CSCs

CSCs represent a subpopulation of cells in solid tumors that drive tumor initiation and progression due to their ability to self-renew and generate differentiated malignant progeny^34^,^35^. To test whether CNT-induced CAF-like cells could promote tumor formation through the activation of a CSC niche, we co-cultured CNT-induced CAF-like cells with human lung carcinoma H460 cells and analyzed CSC generation by measuring tumor sphere formation, a functional assay for CSC formation under selective condition of serum-free media and non-adherence, and side population (SP). To determine the effect of microenvironment on CSC growth, we first tested the influence of LFs on CSC tumor spheres. In accordance with previous *in vivo* tumor formation results, the presence of untreated LF cells was shown to support the growth of lung CSC spheres (Supplementary Figure S2B). While we found that CNT-induced CAF-like cells alone minimally survived (Supplementary Figure S2C), co-culture of them (LF/SWCNT and LF/f-MWCNT) with lung carcinoma H460 cells resulted in a greater number of large tumor spheres when compared to the co-culture of H460 cells and control LF/BSA cells (Fig. 3B).

**Figure 4:**
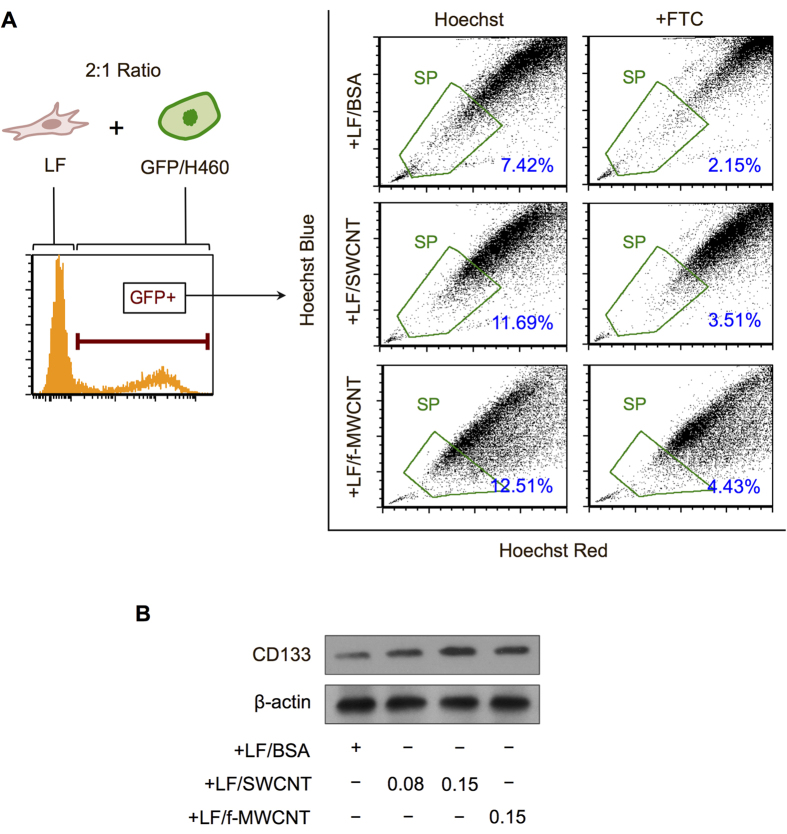
Analysis of side population and lung stem cell marker CD133 in lung carcinoma cells cultured with carbon nanotube-induced cancer-associated fibroblast-like cells. Carbon nanotube-induced cancer-associated fibroblast-like cells (LF/SWCNT or LF/f-MWCNT) or vehicle-treated fibroblasts (LF/BSA) were co-cultured with GFP-labeled lung cancer H460 cells (2:1 ratio). (**A**) Analysis of side population (SP) of the GFP-labeled H460 cells in the presence or absence of fumitremorgin c (FTC) using FACS. SP cells (*box*) are determined by their disappearance in the presence of FTC. (**B**) Western blot analysis of lung stem cell marker CD133 in the co-culture of H460 cells with LF/BSA, LF/SWCNT or LF/f-MWCNT cells.

Likewise, SP analysis, which is a flow cytometric measurement commonly used to assess of the ability of CSCs to efflux Hoechst 33342 dye due to their high ABCG2 expression^36^,^37^, indicated an increase in the CSC proportion in the H460 cell population, which have been modified to express green fluorescence protein (GFP) to aid their identification, when co-cultured with CNT-induced CAF-like cells (LF/SWCNT and LF/f-MWCNT) as compared to control (LF/BSA) (Fig. 4A). We also observed an increase in lung CSC marker CD133 level in the co-culture of H460 cells and CNT-induced CAF-like cells (Fig. 4B). Together, these results indicate the ability of CNT-induced CAF-like cells to provide a supportive microenvironment for CSC growth and maintenance.

### Podoplanin as a mechanism-based biomarker for CNT tumorigenicity

**Figure 5:**
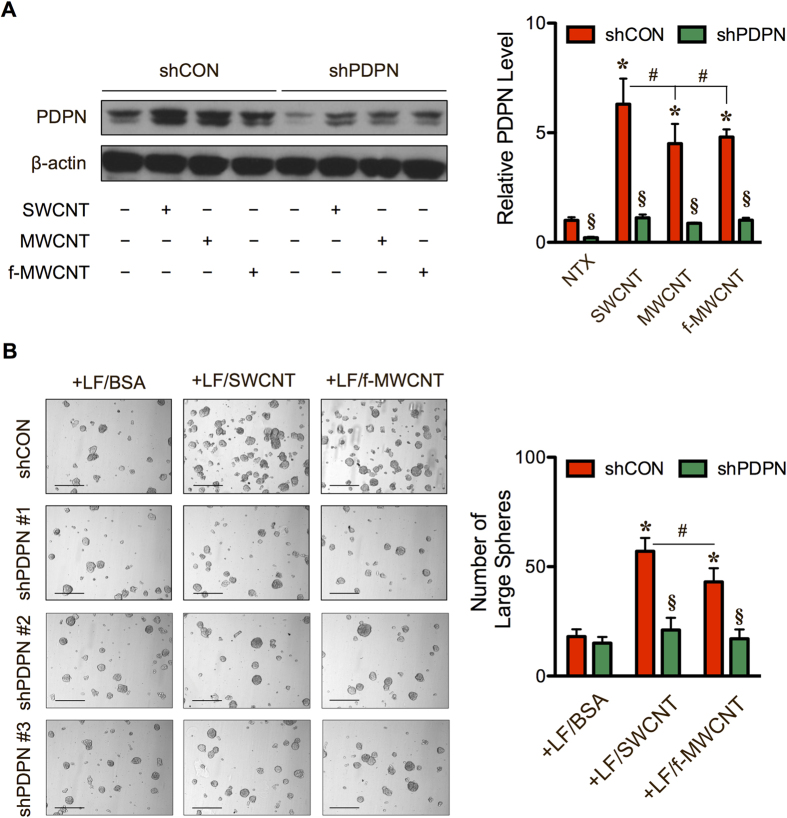
Carbon nanotube-induced cancer associated fibroblast-like cells are incapable of inducing cancer stem cells in the absence of podoplanin. (**A**) Human lung fibroblasts were stably transfected with shRNA plasmid against podoplanin (shPDPN) or scramble control (shCON), and treated with SWCNT, MWCNT or f-MWCNT at the dose of 0.15 μg/cm^2^ for 48 h. Podoplanin level was detected by Western blotting. (**B**) Carbon nanotube-induced cancer-associated shCON fibroblast-like cells (shCON-LF/SWCNT or LF/f-MWCNT) or carbon nanotube-treated shPDPN fibroblast-like cells (shPDPN-LF/SWNCT or LF/f-MWCNT) were co-cultured with H460 cells (2:1 ratio), and tumor sphere formation was analyzed after 2 weeks of culture. Scale bar = 200 μm. Data are mean ± SD (*n* = 4). *P < 0.05 *vs* H460 and shCON-LF/BSA group. ^§^*P* < 0.05 *vs* CNT-treated shCON group. ^#^*P* < 0.05 *vs* shCON-LF/SWCNT group.

**Figure 6:**
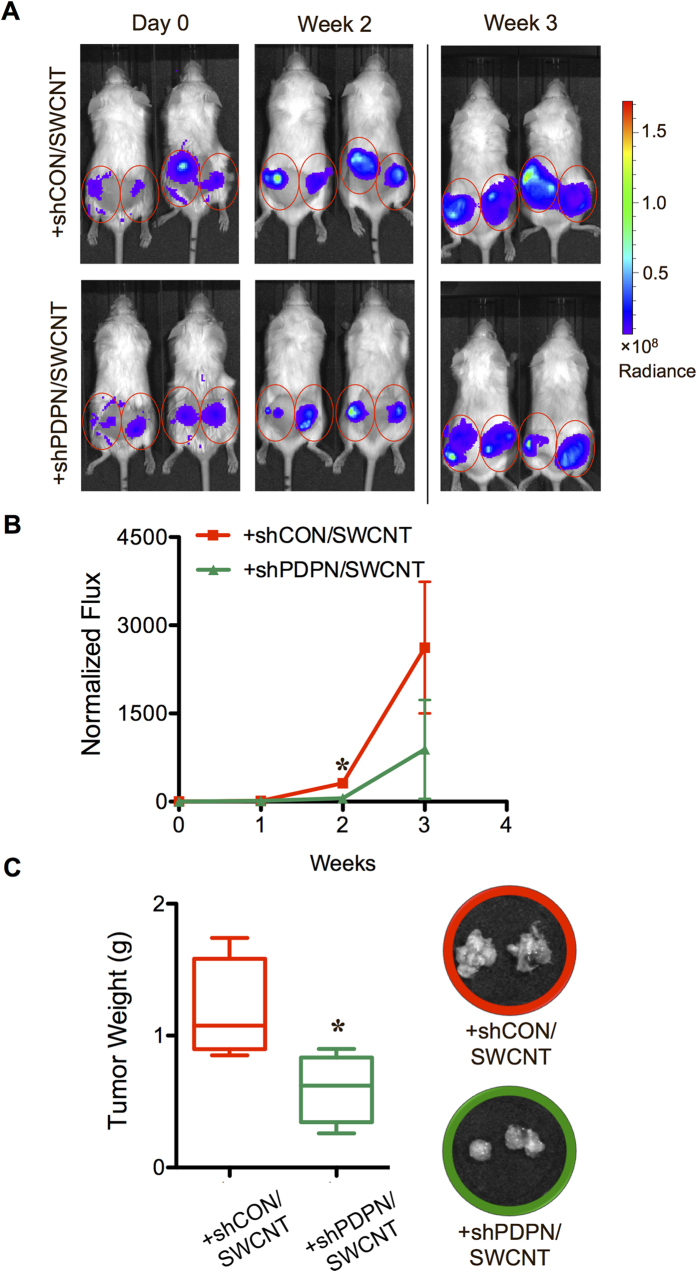
Podoplanin is required for the tumor promoting effect of carbon nanotube-induced cancer-associated fibroblasts. (**A**) Carbon nanotube-induced cancer-associated shCON fibroblast-like cells (shCON/SWCNT) or carbon nanotube-treated shPDPN fibroblast-like cells (shPDPN/SWCNT) at the dose of 6 × 10^5^ cells were co-injected with luciferase-labeled H460 cells at the dose of 3 × 10^5^ cells (2:1 ratio) into the left and right flanks of NSG mice. Tumor formation was monitored weekly by IVIS bioluminescence imaging. IVIS images of mice at day 0 and week 2 and 3 are shown. (**B**) Normalization of tumor bioluminescence signals at various time points post-injection to their initial signals at day 0. (**C**) Subcutaneous tumors were dissected from mice bearing H460 and LF/SWCNT or LF/BSA, and were weighted at the end of experiments at 3 weeks post-injection. Data are mean ± SD (*n* = 4). *P < 0.05 *vs* H460 and shCON/SWCNT group.

To further investigate the role of CNT-induced CAF-like cells in the tumor formation of lung cancer cells and to develop a potential predictive biomarker for CNT carcinogenicity, we evaluated the functional role of podoplanin in the tumor-promoting effect of CNT-induced CAF-like cells. Podoplanin, also known as Gp36, is a transmembrane siaglycoprotein that is expressed in CAFs of cancerous stroma and has been identified as a new marker of CAFs in various cancers, including lung^19^,^38^. Primary human LFs were stably transfected with short-hairpin (sh) RNA plasmid against podoplanin (shPDPN) or control vector (shCON), and their effects on CSC formation and tumorigenesis of lung cancer cells were examined. Figure 5A shows that the shPDPN-transfected fibroblasts expressed a substantially lower level of both basal and CNT-induced podoplanin expression than the shCON-transfected cells. These knockdown and control cells were used to examine the functional role of podoplanin in CSC formation and tumorigenesis by *in vitro* tumor sphere formation and *in vivo* xenograft tumor formation assays, respectively. Figure 5B shows that the podoplanin-knockdown cells, either CNT-treated (shPDPN-LF/SWCNT, shPDPN-LF/f-MWCNT) or vehicle-treated (shPDPN-LF/BSA), were relatively ineffective in inducing CSCs from the human lung carcinoma H460 cells, as compared to vector-transfected control cells (shCON-LF/SWCNT, shCON-LF/f-MWCNT). Likewise, *in vivo* tumorigenesis studies showed that co-injection of CNT-treated podoplanin-knockdown cells with lung carcinoma H460 cells yielded a lower level of tumor-associated luminescence signals (Fig. 6A and B). Direct measurements of the tumor weight from the treated animals confirmed the finding (Fig. 6C). These results indicate that podoplanin is crucial to the tumor-promoting effect of CNT-induced CAF-like cells, and their CSC-enhancing activity. These results also suggest that podoplanin may be used as a potential biomarker for CNT carcinogenicity.

### CNTs provoke a specific CSC niche for lung tumorigenicity

**Figure 7:**
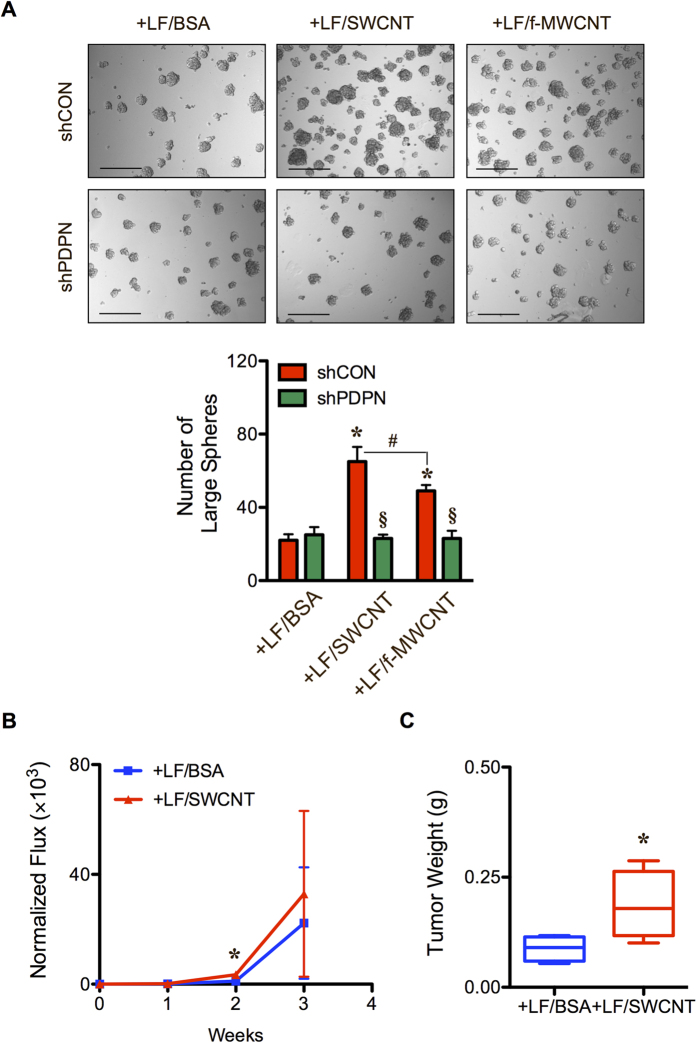
Carbon nanotube-induced cancer-associated fibroblast-like cells and podoplanin level are associated with tumorigenicity of carbon nanotube-transformed cells. (**A**) Carbon nanotube-induced cancer-associated shCON fibroblast-like cells (shCON-LF/SWCNT or LF/f-MWCNT) or carbon nanotube-treated shPDPN fibroblast-like cells (shPDPN-LF/SWCNT or LF/f-MWCNT) were co-cultured with SWCNT-transformed lung epithelial BEAS-2B/SWCNT cells (2:1 ratio), and tumor sphere formation was analyzed after 2 weeks of culture. Scale bar = 200 μm. (**B**) Carbon nanotube-induced cancer-associated fibroblast-like cells (LF/SWCNT) or vehicle-treated fibroblasts (LF/BSA) were co-injected with SWCNT-transformed lung epithelial BEAS-2B/SWNCT cells (2:1 ratio) into the left and right flanks of NSG mice. Tumor formation was monitored weekly by IVIS bioluminescence imaging. Normalization of tumor bioluminescence signals at various time points post-injection to their initial signals at day 0. (**C**) Subcutaneous tumors were dissected from mice bearing BEAS-2B/SWCNT and LF/SWCNT or LF/BSA, and were weighted at the end of experiments at 3 weeks post-injection. Data are mean ± SD (*n* = 4). *P < 0.05 *vs* H460 and shCON-LF/BSA group. ^§^*P* < 0.05 *vs* CNT-treated shCON group. ^#^*P* < 0.05 *vs* shCON-LF/SWCNT group.

Increasing evidence suggests that certain types of CNTs are carcinogenic^7^,^8^,^9^,^10^,^11^,^12^,^39^,^40^. Although the mechanism underlying this carcinogenicity remains incompletely understood, an induction of CSCs by CNTs has been implicated^13^,^14^. Based on our finding in the present study showing the induction of CSCs from human lung carcinoma cells by CNT-induced CAF-like cells, we further investigated whether these CAFs can also induce CSCs from CNT-transformed human lung epithelial cells, and whether or not such induction is dependent on podoplanin. First, CNT-induced CAF-like cells (LF/SWCNT and LF/f-MWCNT) were co-cultured with human lung epithelial cells that had been previously transformed by chronic exposure to CNTs (BEAS-2B/SWCNT) as previously described^13^,^14^,^40^. These CNT-transformed cells were shown to be tumorigenic and contain CSC subpopulation^13^,^14^, which were further investigated in this study to examine the impact of CAF-like cells on their CSC- and tumor-forming capacities. Figure 7A shows that CNT-induced CAF-like cells indeed increased the level of CSC subpopulation in the transformed epithelial cell population as determined by tumor sphere formation assay. Interestingly, knockdown of podoplanin in the CNT-induced CAF-like cells completely inhibited their effect on CSC formation (Fig. 7A), consistent with the earlier finding in H460 cells and supporting the functional role of podoplanin in CSC regulation. Next, we determined the effect of CNT-induced CAF-like cells on tumor formation of CNT-transformed lung epithelial (BEAS-2B/SWCNT) cells in mice. Figure 7B and C shows that the CAFs were able to promote tumor growth of the CNT-transformed lung epithelial cells. These results indicate that CNTs are capable of not only transforming lung epithelial cells to become tumorigenic but also altering their microenvironment by inducing CAF-like cells that support tumor growth.

## Discussion

Lung cancer is the leading cause of cancer mortality and is strongly associated with environmental and occupational exposure^41^,^42^. Due to their extremely small size and respirable nature, nanoparticles are a prime candidate for lung cancer risk. Analysis of environmental nanoparticles revealed that there could be between 20,000–100,000 nanoparticles/cm^3^ in the atmosphere, with a significant fraction being CNTs^43^,^44^. Considering concerns over the increasing utility of CNTs and their potential health hazards, the objective of this study was to investigate cancer-related pathologies and the underlying mechanisms in order to develop biomarkers and rapid assessment tools for carcinogenicity testing of nanomaterials and to aid safe-by-design efforts. In the context of carcinogenesis, any substances that act on one or more stages of tumor initiation, promotion and progression are considered carcinogens^45^,^46^. Carcinogens exert their effects on cells and tissues in two ways: (i) direct effects on target cells, i.e., by causing DNA mutation and oncogene activation; and (ii) indirect effects on neighboring cells that comprise the tumor microenvironment and are required for tumor growth and development. These two mechanisms generally work in concert to produce the carcinogenic effect caused by an agent. With regard to CNTs, we have previously demonstrated that chronic exposure to certain CNTs induced malignant transformation and tumorigenesis of human lung epithelial cells^13^,^14^,^39^,^40^. However, the role of tumor microenvironment and the mechanism by which it regulates the tumorigenesis are not known. In this study, we demonstrated for the first time that CNTs, notably SWCNT and to a lesser extent MWCNT and f-MWCNT, activated LFs to become CAF-like cells, which are the key component of the tumor microenvironment that supports tumor growth (Fig. 1). These CNT-induced CAF-like cells were shown to promote tumor formation of human lung carcinoma H460 cells (Fig. 2) and CNT-transformed lung epithelial cells (Fig. 7) in a xenograft mouse model. These cells, however, did not induce tumor formation on their own, indicating the supporting role of CAF-like cells in the tumor development (data not shown). These results are consistent with the earlier reports showing the modulation of the tumor microenvironment by CNTs that promotes tumor growth. For example, a single pharyngeal aspiration of SWCNT in mice was shown to induce an accumulation of myeloid-derived suppressor cells in the lungs that resulted in host immunosuppression and lung carcinoma acceleration^7^,^8^. The detection of CNT-induced CAF-like cells could be beneficial over the previously reported CNT-induced lung epithelial cell transformation in view of its rapid response to CNT stimuli (e.g. within 48 h), making it a feasible method for pre-screening of possible CNT carcinogens for further detailed analyses, e.g. in chronic exposure studies.

It is widely accepted that certain physicochemical properties affect CNT bioactivities, including fibroblastic responses. The greater capability of SWCNT to activate CAF-like cells over MWCNT in the present study is consistent with previous studies indicating that SWCNT induced greater interstitial pulmonary fibrosis and collagen deposition *in vivo*^47^,^48^ and induced higher fibroblast stem-like cells and 3D-fibroblastic nodules that resembled clinical fibroblastic foci *in vitro*^24^. With respect to functionalization of CNTs, conflicting results have been reported. While several studies indicated that COOH functionalization significantly decreased CNT bioactivities, e.g. fibrogenic responses and pulmonary inflammation^49^,^50^,^51^,^52^, we and some others observed an increase in cytogenic, genotoxic and fibrogenic activities by COOH functionalization^53^,^54^. In the present study, f-MWCNT elicited more robust effects on CAF-like cells and CSCs as compared to MWCNT. Thus, the relative potency is summarized as SWCNT >f-MWCNT >MWCNT. Notably, variations in experimental conditions and assay methods may account for the observed inconsistencies and more experimental data on the *in vivo* CNT carcinogenicity are needed to substantiate these effects. In addition to affecting normal tumor cells, increasing evidence also indicates that the tumor microenvironment regulates CSCs that drive tumorigenesis. In fact, the severity or aggressiveness of specific cancer types, including lung cancer, is frequently associated with their CSC population^14^,^34^,^36^. Furthermore, the carcinogenicity of several environmental and occupational carcinogens, such as tobacco, arsenic, and CNTs, has been linked to their CSC inducing capability^13^,^55^,^56^. In this study, we observed an induction of CSCs by CNT-induced CAF-like cells (Fig. 3), supporting the role of these CAF-like cells in the CSC growth and maintenance. This finding is consistent with other recent reports showing the importance of CAFs in controlling the plasticity of cancer stemness^18^,^57^. CAFs were shown to regulate the growth of CSCs in a paracrine manner via the insulin-like growth factor (IGF)-II/IGF1 receptor (IGF1R)/Nanog signaling pathway^18^. Interestingly, we found that CNT-induced CAF-like cells promoted the growth of CNT-transformed lung epithelial cells and their CSC subpopulation (Fig. 7), indicating that CNTs are capable of exerting multiple effects on different cell types, i.e., by causing transformation of normal LFs into CAF-like cells, lung epithelial cells into malignant cells, and by regulating CSCs that could serve as a continuous source of cancer cells.

One of the key goals of this study was to develop a mechanism-based biomarker for carcinogenicity testing and risk assessment of nanomaterials. We identified podoplanin as a potential biomarker based on its robust upregulation by CNTs and its functional role in tumorigenesis. Downregulation of podoplanin in LFs by RNA interference strongly inhibited CNT-induced CAF-like cells and their effects on CSCs and subsequent tumor formation (Figs 5, 6 and 7), providing new insights into the role of podoplanin in CSC growth and maintenance mediated by CAF-like cells. Our findings also highlight the importance of podoplanin as a predictive biomarker for CNT-induced carcinogenesis. Increasing evidence indicates that podoplanin has a prognostic significance for different types of tumors^38^. In lung cancer, the presence of podoplanin-positive CAFs predicted advanced pathological stage, e.g. the presence of lymph node metastasis as well as vascular and pleural invasion, and poor outcome among patients with adenocarcinoma and squamous cell carcinoma^58^,^59^,^60^. A recent findings showed that podoplanin assisted lung tumor cells with local invasion; that is tumor cells invaded by following the podoplanin-positive CAFs into surrounding extracellular matrix through RhoA activation^61^. It is worth noting that podoplanin expression was associated with a history of smoking^38^, which might be linked to the mechanism of lung carcinogenesis. The potential advantages of podoplanin as a biomarker include: (i) its rapidity of detection, i.e., the induction of podoplanin by CNTs can be detected within 48 h after exposure; (ii) potential for high-throughput screening assay allowing analysis of a large number of samples with a fewer number of cells per assay, i.e., by using high-content imaging or in-cell Western assays; and (iii) its functional importance and clinical relevance, i.e., podoplanin expression in CAFs has been shown to correlate with poor prognosis and decreased survival of lung cancer patients as earlier mentioned.

**Figure 8:**
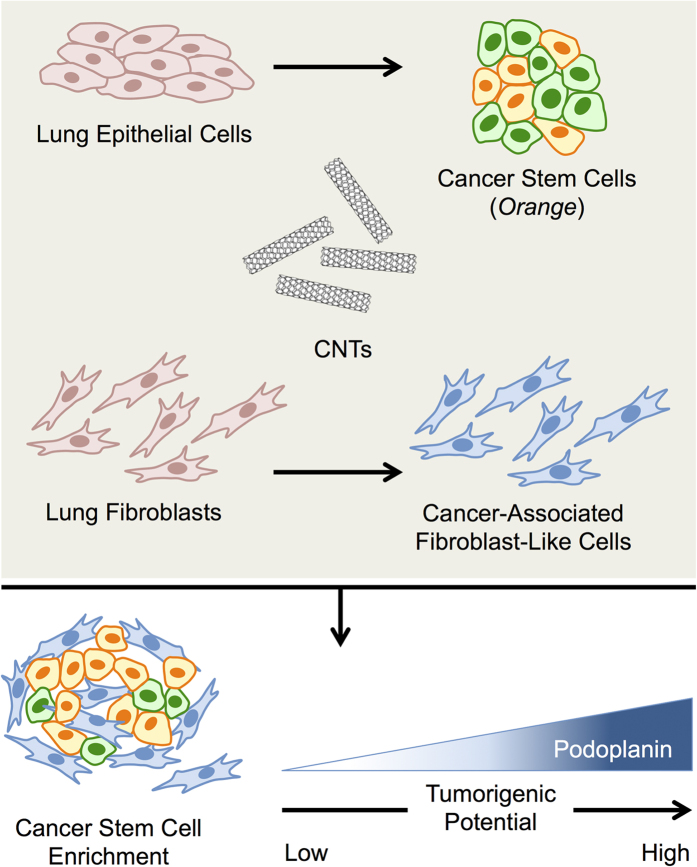
A schematic working model for the roles of carbon nanotubes in tumor promotion. Carbon nanotubes can induce cancer associated fibroblast-like cells that promote cancer stem cell formation in lung epithelial cells.

In conclusion, we have provided novel evidence, as schematically summarized in Fig. 8, that CNTs can induce CAF-like cells that promote CSC formation in lung epithelial cells. Since CSCs are shown to be a key driver of tumorigenesis, our finding concerning CSC induction by CNT-derived CAF-like cells offers a new insight into the possible mechanism of CNT-induced carcinogenesis. Due to its elevated expression and requirement for CSC induction and tumorigenesis, we suggest that podoplanin may be used as a predictive biomarker for cancer risk assessment and for safer by design screening of CNTs and related nanomaterials.

## Materials and Methods

### Cell culture and CNT exposure

Primary normal human LFs were obtained from Lonza (Walkersville, MD) and were cultured in FGM-2 medium containing 2% fetal bovine serum (FBS), 0.1% human basic fibroblast growth factor, 0.1% insulin and 0.1% gentamicin (Lonza). Early-passage cells (p < 6) were seeded (5 × 10^5^ cells/well) in 6-well plate and exposed to lightly sonicated, BSA-dispersed CNTs (Cheap Tubes Inc.), including SWCNT, MWCNT and f-MWCNT at various concentrations for 48 h. Vehicle-only exposed cells were used as a control. Non-small lung cancer cell (NSCLC)-H460 cells were obtained from American Type Culture Collection (ATCC; Manassas, VA) and were cultured in RPMI 1640 medium supplemented with 5% FBS, 2 mM L-glutamine, and 100 units/mL penicillin and 100 μg/mL streptomycin (P/S). Human bronchial epithelial BEAS-2B cells were obtained from ATCC and were chronically exposed to low-dose SWCNT (0.02 μg/cm^2^) for 6 months to generate BEAS-2B/SWCNT cells as previously described^13^,^14^,^40^. They were cultured in Dulbecco’s modified Eagle medium (DMEM) supplemented with 5% FBS, 2 mM L-glutamine and P/S. All cells were maintained in a humidified atmosphere of 5% CO_2_ at 37 °C.

### Cell viability assay

Cell viability was determined using WST-1 assay kit (Roche Molecular Biochemicals, Indianapolis, IN, USA) following the manufacturer’s instructions. Cells were seeded (1 × 10^4^ cells/well) in quadruplicate in 96-well plates in culture medium and were exposed to various concentrations (0–0.15 μg/cm^2^) of SWCNT, MWCNT or f-MWCNT for 48 h. The cells were then incubated with WST-1 reagent and incubated for an additional 4 h. The absorbance was measured at 450 nm using a microplate reader (FLUOstar OPTIMA, BMG Labtech, Durham, NC) and relative cell viability was calculated by dividing the absorbance of the treated cells by that of the control (non-treated) cells.

### Western blot analysis

After specific treatments, cells were incubated in a commercialized lysis buffer (Cell Signaling Technology, Beverly, MA) and a protease inhibitor mixture (Roche Molecular Biochemicals, Indianapolis, IN) at 4 °C for 20 min. The cell lysates were collected and determined for protein content using the Pierce BCA protein assay (Pierce Biotechnology, Rockford, IL). Proteins (40 μg) were resolved under denaturing conditions by 12% sodium dodecyl sulfate-polyacrylamide gel electrophoresis (SDS-PAGE) and transferred onto PVDF membranes (Invitrogen, Carlsbad, CA). The transferred membranes were blocked for 1 h in 5% nonfat dry milk in TBST (25 mM Tris–HCl, pH 7.4, 125 mM NaCl, 0.05% Tween 20) and incubated with the appropriate primary antibodies, i.e. anti-podoplanin (ABT34; Millipore, Billerica, MA) or anti-α-SMA (ab5694; Abcam, Cambridge, MA), at 4 °C overnight. Membranes were washed twice with TBST for 10 min and incubated with horseradish peroxidase-coupled isotype-specific secondary antibodies for 1 h at room temperature. The immune complexes were detected by an enhanced chemiluminescence detection system (Amersham Biosciences, Piscataway, NJ) and quantified using analyst/PC densitometry software (Bio-Rad Laboratories, Hercules, CA).

### TGF-β enzyme-linked immunosorbent assay (ELISA)

Cells were seeded (2 × 10^5^ cells/well) in triplicate in 6-well plates in culture medium and were exposed to various concentrations (0–0.15 μg/cm^2^) of SWCNT, MWCNT or f-MWCNT for 48 h. Cell supernatants were then collected and analyzed for TGF-β by ELISA using a commercial kit (R&D Systems, MN). Briefly, cell supernatant samples or reference standards (100 μL) were added to the wells of a microplate that was pre-coated with TGF-β monoclonal antibody and incubated for 2 h at room temperature. After washing unbounded substances, a HRP-conjugated polyclonal antibody against TGF- β was added to the wells and incubated for 2 h at room temperature. After washing and addition of 100 μL of substrate solution, optical density was determined on a microplate reader (FLUOstar OPTIMA BMG LABTECH Inc., Durham, NC, USA) at 450 nm.

### Xenograft mouse model

Animal care and experimental procedures described in this study were performed in accordance with the Guidelines for Animal Experiments at West Virginia University and were approved by the Institutional Animal Care and Use Committee (IACUC #12–0502). Immunodeficient NOD/SCID gamma mice, strain NOD.Cg-Prkdc^scid^ Il2rg^tm1Wjl^/SzJ (NSG; Animal Models and Imagining Facility, Mary Babb Randolph Cancer Center, Morgantown, WV), were maintained under pathogen-free conditions within the institutional animal facility^62^. Food and tap water were given ad libitum. Mice were subcutaneously injected with 6 × 10^5^ CNT-exposed LFs and 3 × 10^5^ luciferase (LUC2)-labeled H460 or BEAS-2B/SWCNT cells (2:1 ratio) suspended in 100 μL of ExtraCel hydrogel (Advanced BioMatrix, San Diego, CA). Tumor growth of luciferase-labeled cells was monitored weekly using IVIS bioimaging system (PerkinElmer, Waltham, MA). For quantitative comparison, normalized flux was calculated by diving the luminescence flux signal at a given time by that of the signal at the time of inoculation. At the end of experiments, mice were euthanized by carbon dioxide inhalation, and subcutaneous tumors were dissected and weighted.

### Plasmids and transfection

GFP plasmid was obtained from Invitrogen (Carlsbad, CA), while shRNA plasmids against human PDPN (shPDPN) and scrambled vector (shCON) were obtained from SABiosciences (SureSilencing^TM^, Frederick, MD). For transfection, 1 × 10^6^ cells were suspended in 100 μL of nucleofection solution with 2 μg of plasmid and nucleofected using Nucleofector^®^ (Amexa Biosystems, Cologne, Germany) with the device program T020 for H460 and BEAS/SWCNT and program U 023 for LFs. The cells were then resuspended in 500 μL of complete medium and seeded in 60-mm cell culture dishes. The cells were allowed to recover for 48 h before each experiment and were sorted for GFP-positive cells using a BD FACSAria cell sorter (BD Biosciences, San Jose, CA).

### SP analysis

Cells were detached by trypsinization, and 1 × 10^6^ cells were labeled with 5 μg/mL of Hoechst 33342 in DMEM-F12 medium containing 2% FBS in the presence or absence of 25 μM ABCG2 inhibitor fumitremorgin C (FTC; EMD Biosciences, San Diego, CA) at 37 °C for 90 min. The cells were then centrifuged and resuspended in ice-cold Hank’s buffer salt solution (HBSS). SP analysis and sorting were performed using a BD FACSAria cell sorter (BD Biosciences, Franklin Lakes, NJ). The Hoechst dye was excited with a UV laser and its fluorescence was measured with both a 450/20 filter (Hoechst Blue) and 675 LP filter (Hoechst Red). The SP fraction was calculated based on the disappearance of SP cells in the presence of FTC using the formula: SP percentage in the absence of FTC − SP percentage in the presence of FTC.

### Soft agar colony formation

Soft agar assay was performed as previously described^13^. CNT-exposed LFs (2 × 10^4^ cells) and H460 or BEAS-2B/SWCNT cells (1 × 10^4^ cells) were mixed with culture medium containing 0.5% agar to a final agar concentration of 0.33% at 2:1 ratio. Cell suspensions were immediately plated onto dishes coated with 0.5% agar in culture medium. Large (>50 μm) colonies were examined under a light microscope after two weeks of culture.

### Tumor sphere assay

Tumor sphere assay was performed under non-adherent and serum-free conditions as previously described as stem cell-selective conditions^37^. Briefly, 1 × 10^4^ CNT-exposed LFs and 5 × 10^3^ H460 or BEAS-2B/SWCNT cells (2:1 ratio) were resuspended in 0.8% methylcellulose (MC)-based serum-free medium (Stem Cell Technologies, Vancouver, Canada) supplemented with 20 ng/mL epidermal growth factor (BD Biosciences), basic fibroblast growth factor and 4 mg/mL insulin (Sigma). The cell suspension was plated in ultralow adherent 24-well plates (Corning, Corning, NY) and cultured for two weeks. Large (>50 μm) tumor spheres were quantified under a light microscope.

### Statistical analysis

The data represent means ± SD from three or more independent experiments as indicated. Statistical analysis was performed by Student’s t test at a significance level of *P* < 0.05. An ANOVA followed by Mann-Whitney U test was used for a multiple pairwise comparison.

## Additional Information

**How to cite this article**: Luanpitpong, S. *et al*. Induction of cancer-associated fibroblast-like cells by carbon nanotubes dictates its tumorigenicity. *Sci. Rep.* **6**, 39558; doi: 10.1038/srep39558 (2016).

**Publisher's note:** Springer Nature remains neutral with regard to jurisdictional claims in published maps and institutional affiliations.

## Supplementary Material

Supplementary Information

## Figures and Tables

**Table 1:** Physicochemical properties of carbon nanotubes used in this study.

	SWCNT	MWCNT	f-MWCNT
Source	Cheap Tubes Inc.	Cheap Tubes Inc.	Cheap Tubes Inc.
Catalog reference	SKU 0111	SKU 030111	SKU 030113
Synthesis method^a^	CCVD	CCVD	CCVD
Primary functionality^a^	Pristine	Pristine	COOH (1.8%)
Dry mean width (nm)^a^	1–2	13–18	13–18
Dry mean length (μm)^a^	5–30	1–12	1–12
% elemental carbon^a^	>95%	>95%	>95%
% iron impurity^b^	0.12%	Not detectable	N/A
Other metal impurities^b^	Cr 0.31%	Ni 1.88%	N/A
	Co 0.1%	Al 0.04%	
	Si 0.08%	Si 0.03%	
SSA (m^2^/g)^a^	>407	>233	>233
Zeta potential (mV)^c^	−7.44	−9.58	−8.66

^a^Data from manufacturer’s datasheet.

^b^Data from in-house analysis using energy dispersive X-ray spectroscopy (EDX-S).

^c^Data from in-house analysis using Nano Series ZetaSizer. CCVD, plasma purified catalytic chemical vapor deposition. SSA, specific surface area.
