# Supplementary material for: Induction of cancer-associated fibroblast-like cells by carbon nanotubes dictates its tumorigenicity
Source: Sci Rep. 2016 Dec 20;6:39558. doi: 10.1038/srep39558 (PMC5172236; doi:10.1038/srep39558)
Supplement: Supplementary Information [file srep39558-s1.pdf]

Induction of cancer-associated fibroblast-like cells by carbon nanotubes dictates its tumorigenicity

Sudjit Luanpitpong[1,2,*], Liying Wang[3], Vincent Castranova[2], Cerasela Zoica Dinu[4], Surapol Issaragrisil[1], Yi Charlie Chen[5], Yon Rojanasakul[2,6,*]

[1]Siriraj Center of Excellence for Stem Cell Research, Faculty of Medicine Siriraj Hospital, Mahidol University, Bangkok 10700, Thailand

[2]Pharmaceutical and Pharmacological Sciences Program, West Virginia University, WV 26506, USA

[3]Allergy and Clinical Immunology Branch, National Institute for Occupational Safety and Health, Morgantown, WV 26505, USA

[4]Department of Chemical Engineering, West Virginia University, WV 26506, USA

[5]Natural Science Division, Alderson Broaddus University, Philippi, WV 26416, USA

[6]West Virginia University Cancer Institute, West Virginia University, WV 26506, USA

Correspondence to: Sudjit Luanpitpong, email: suidjit@gmail.com; Yon Rojanasakul, email: yrojan@hsc.wvu.edu

Keywords: cancer-associated fibroblasts, cancer stem cells, podoplanin, carbon nanotubes, tumorigenesis

Number of supplementary figures: 2

Supplementary information

Supplementary information includes Supplementary Figures S1 and S2.

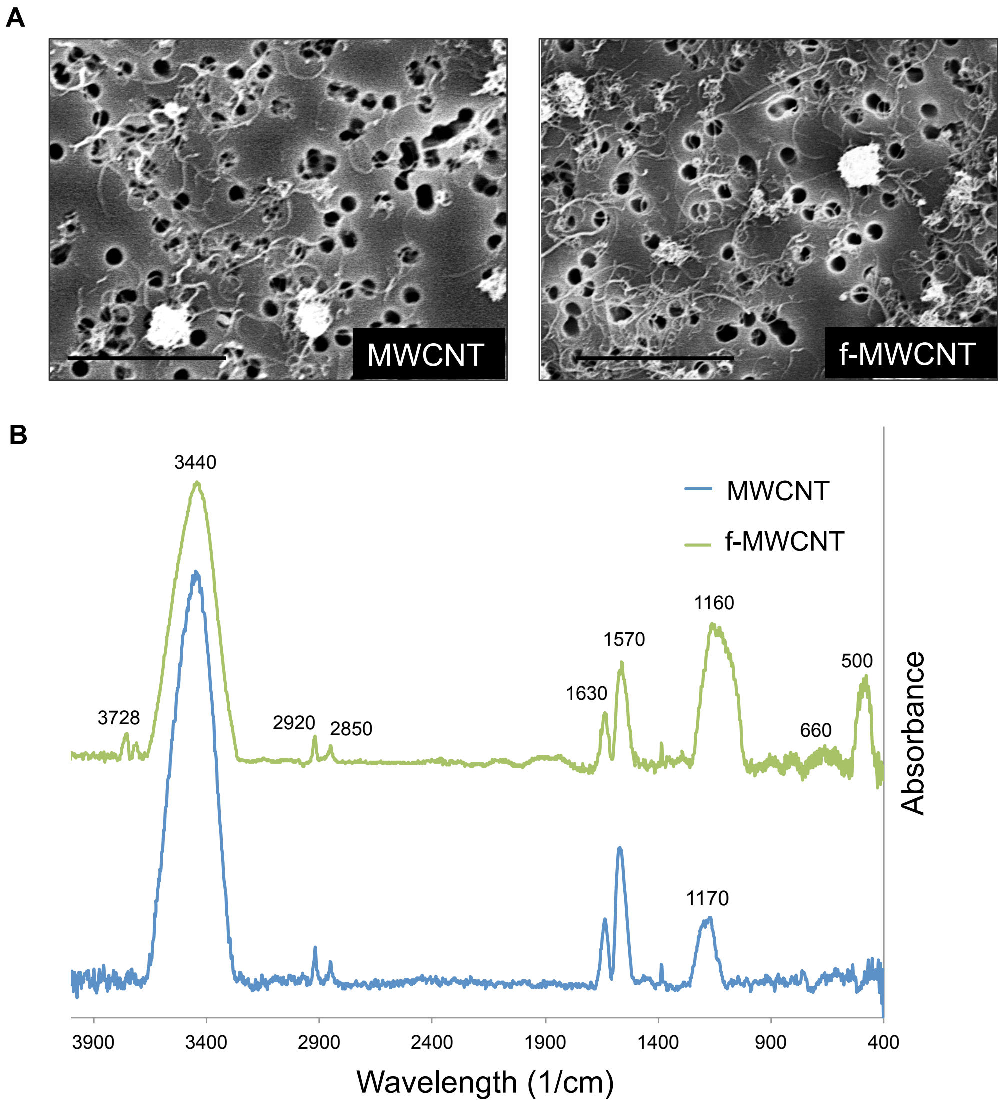

Supplementary Figure S1. Physicochemical characterization of CNTs used in the present study. (*A*) Scanning electron micrographs of dispersed particles. Dispersed MWCNT and f-MWCNT exhibited mostly single fibers and some micro-sized agglomerates. Scale bar = 2 µm. (*B*) Solid state Fourier transform infrared spectroscopy (FTIR) of MWCNT and carboxylate (COOH) f-MWCNT. f-MWCNT shows the typical IR peaks, where (i) the 3420 cm^{-1} peak is assigned to the O−H stretching vibration; (ii) 2833 cm^{-1} peak and 2912 cm^{-1} peaks are ascribed to the symmetric and asymmetric vibrations of C−H, respectively; whereas (iii) the 1626 cm^{-1} and 1550 cm^{-1} peaks are due to the benzene ring skeleton vibrations; and (iv) 1130 cm^{-1} peak depicts the C−O stretching vibration. Additionally, the distinct peaks of vibrational carboxyl group were observed in f-MWCNT spectrum at the frequencies: (i) 3728 cm^{-1} assigned to O−H stretching (O=C-OH and C−OH); (ii) 660 cm^{-1} assigned to C=O out-of-plane bending; and (iii) 574 cm^{-1} assigned to C−OH torsion.

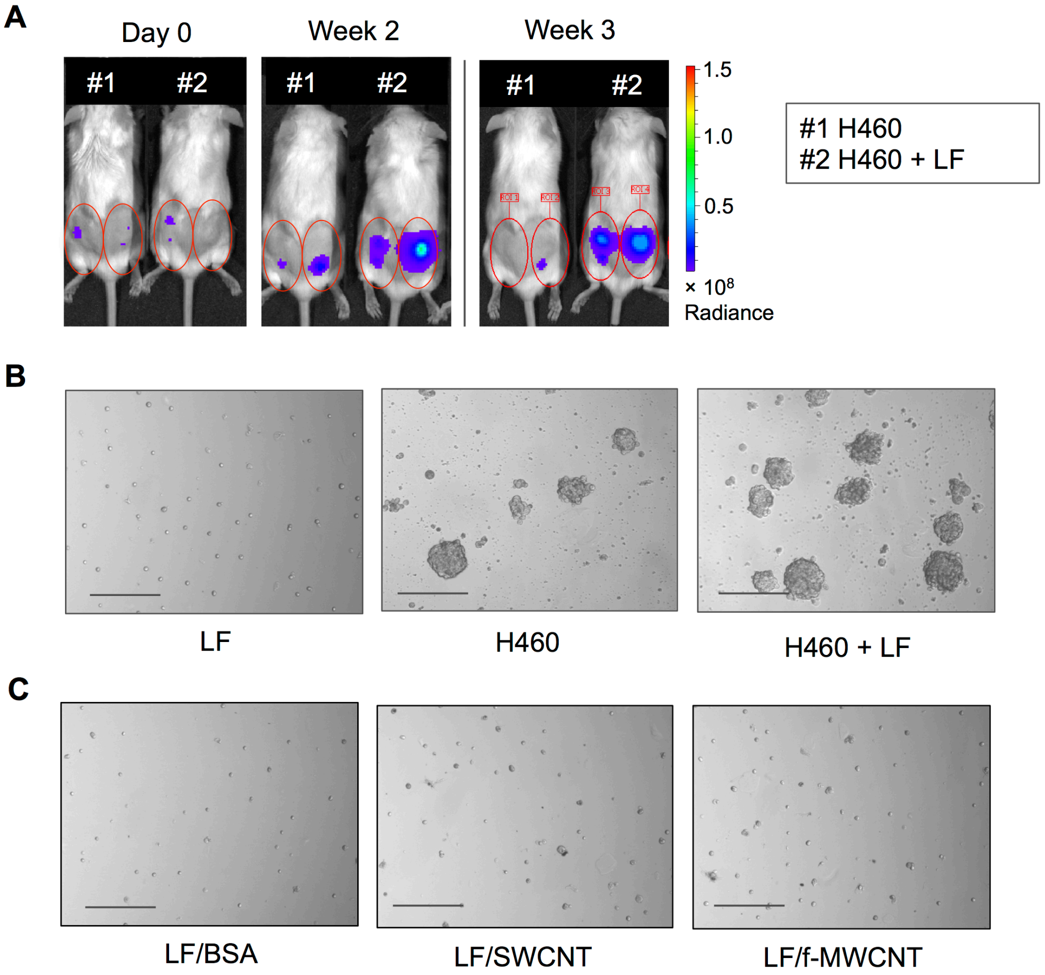

Supplementary Figure S2. Human lung fibroblasts promote tumor formation of human lung carcinoma H460 cells. (*A*) Luciferase-labeled H460 cells at the dose of 3×10^5 cells were SC injected into the left and right flanks of NSG mice in the presence or absence of human lung fibroblasts (LFs) at the dose of 6×10^5 cells. Tumor formation was monitored weekly by IVIS bioluminescence imaging. IVIS images of mice at the time of inoculation (day 0) and week 2 and week 3 are shown. (*B*) Human lung fibroblasts were co-cultured with GFP-labeled H460 cells (2:1 ratio) in methylcellulose-based medium under non-attached, serum-starved conditions. Tumor spheres colonies were analyzed after 2 weeks of culture. Scale bar = 300 µm. (*C*) Analysis of sphere formation of carbon nanotube-induced cancer-associated fibroblast-like cells (LF/SWCNT or LF/f-MWCNT) or vehicle-treated fibroblasts alone after 2 weeks of culture indicates minimal fibroblast cell survival. Scale bar = 300 µm.
